# Ileocecal Valve Atresia – A Take on the Aberrant Phenomenon

**DOI:** 10.7759/cureus.17614

**Published:** 2021-08-31

**Authors:** Anup Kumar Panigrahi, Priyanka Anvekar, Petras Lohana, Mohammed Elmahal, Sherif A Baath Allah

**Affiliations:** 1 Minimal Access Surgery, Safdarjung Hospital and Lohia Hospital, New Delhi, IND; 2 Medicine and Surgery, Mahatma Gandhi Mission (MGM) Medical College and Hospital, Mumbai, IND; 3 Internal Medicine, Liaquat University of Medical and Health Sciences Hospital, Karachi, PAK; 4 Internal Medicine, University of London, London, GBR; 5 Pediatrics, Cairo University, Giza, EGY

**Keywords:** ileocecal valve, appendectomy, intestinal obstruction, pediatric emergency, ileocecal atresia

## Abstract

Ileocecal valve atresia is the most uncommon yet remarkable form of the atresia found within the gastrointestinal system. We report a case on this rare entity with few cases documented in the literature to date. In our case, a one-day-old full-term male infant who developed the signs of intestinal obstruction was eventually taken for emergency laparotomy. The atretic area found intraoperatively was removed followed by the creation of an anastomosis. The patient recovered well postoperatively and continues regular pediatric follow-ups.

## Introduction

Colonic atresia is a very rare form of intestinal obstruction in neonates, presenting with failure to pass meconium, vomiting and abdominal distension with an incidence rate of 1.8 to 15 per 100 live births in the general population [1]. However, atresia of the colon is known to be the most common determinant leading to intestinal obstruction [2].

About half of the intestinal atresia have been documented in the jejunum and ileum [3]. Although about one-third of the intestinal atresia is limited to the distal ileum, a complete absence of ileocaecal junction is very rare [3]. In the current literature, there are very few cases documented for ileocecal atresia [4-9].

We report a case of a one-day-old male infant diagnosed with intestinal atresia that involved the complete ileocecal junction, with no connection between the small and large intestine with the presence of an appendix. We further intend to discuss the reasons for the conclusions that were made intraoperatively and the potential outcome of the patient.

## Case presentation

A one-day-old male neonate presented with abdominal distension, failure to pass meconium, and bilious vomiting. He was born at 38 weeks gestation by normal vaginal delivery to a multiparous 29-year-old woman and weighed 2.550 Kilograms at birth. There was no remarkable family history.

**Figure 1:**
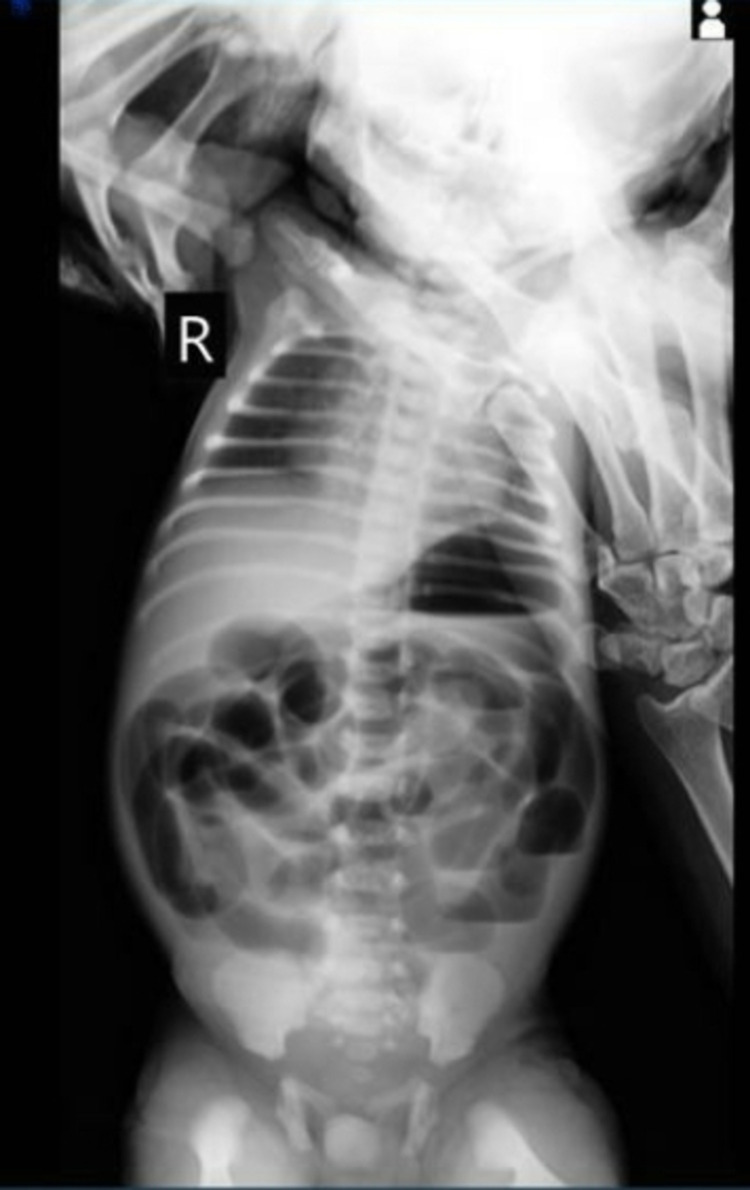
X-ray abdomen showing dilated loops of bowel and absence of bowel gas pattern.

The prenatal ultrasound scan did not reveal any mass fetal anomaly and no evidence of polyhydramnios. An X-ray of the abdomen revealed dilated loops of the bowel with the presence of fluid on the right side and the absence of the bowel gas pattern in the lower abdomen (Figure 1).

Based on X-ray findings, small bowel obstruction was diagnosed, and he was immediately taken for emergency laparotomy.

**Figure 2:**
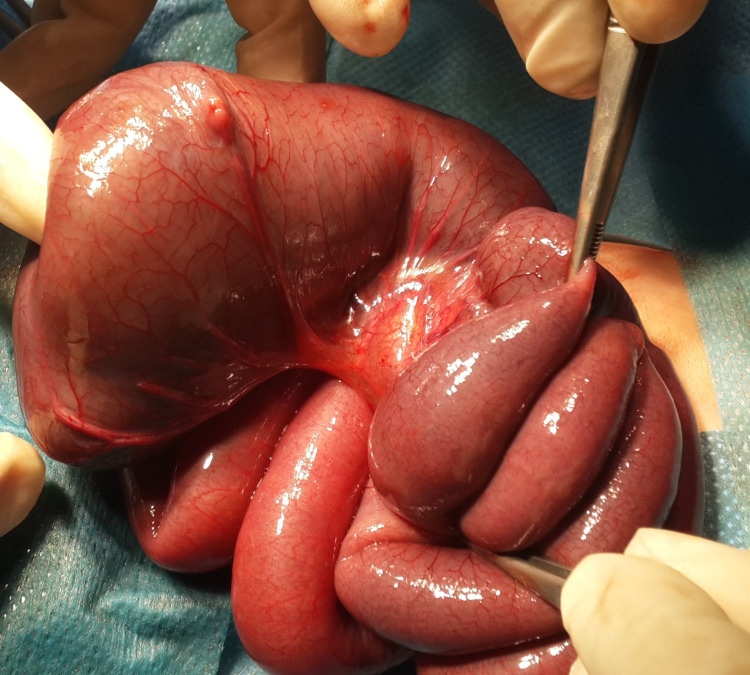
Emergency laparotomy procedure showing distended distal ileum.

**Figure 3:**
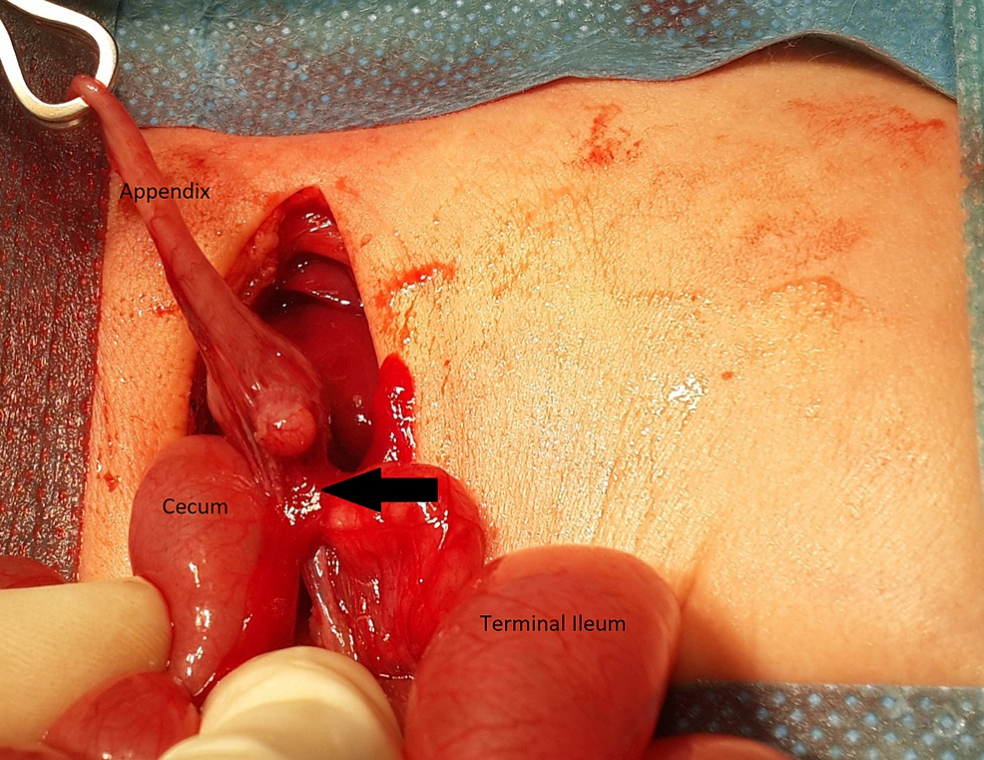
Resection of the atretic part and creation of anastomosis.

During surgery, the right transverse incision in the infraumbilical region was given. The bowel was explored, and it was seen that the terminal ileum was markedly dilated, and the cecum was small and collapsed. There was no connection between the terminal ileum and cecum. The appendix was seen attached to the cecum, which was removed. There was also a V-shaped defect within the mesentery supplying the ileocecal region. We removed the grossly distended last 5 cm of the distal ileum. We performed an end to side ileo ascending anastomosis after confirming the absence of a distal obstruction by irrigating the lumen of ascending colon with normal saline. The mesenteric defect was closed with absorbable sutures (Figures 2-3).

Histopathology of resected ileum showed bowel mucosa with vascular congestion, mild to moderate edematous changes of submucosa and serosa. The appendectomy specimen showed submucous serosal vascular congestion. He had an uneventful hospital course, and the incision was healing well.

He passed meconium on postoperative day 2 and was restarted on oral feeding on a postoperative day 4. He was seen for his one-month follow-up, and examination showed his development on the growth chart was above the 25th percentile and was satisfactory.

## Discussion

Ileocecal atresia is the most uncommon form of colonic atresia. The ileocecal valve creates a tonic pressure blocking the reflux of intestinal contents and also shows a response to various pharmacological and physiologic changes [10]. Atresia can be explained by various etiologies such as the entire ileocecal valve region being undeveloped or under-developed or the obstruction of the valve by the presence of the appendix [5,6]. In our patient, the appendix was noted to be attached to the cecum which acted as a membranous obstruction and can be interpreted as the cause of intestinal obstruction.

In the cases documented in the literature, the neonates present mostly with the signs of intestinal obstruction. The clinical manifestations can include abdominal distension, bilious vomiting, and unable to pass meconium [4-9]. Initially, the ileocecal valve atresia was diagnosed intraoperatively as the preoperative radiologic signs were unknown, however, recently many imaging modalities such as plain X-ray abdomen, upper gastrointestinal contrast study, and contrast enema have been introduced to rule in the disease. A typical finding on the abdominal X-ray would be the presence of dilated loops of the bowel. However, a contrast enema would demonstrate microcolon [11]. In our patient, the abdominal X-ray showed the dilated loops of the bowel with an absent bowel gas pattern to support the diagnosis of intestinal obstruction. Intraoperatively the diagnosis of ileocecal valve atresia was confirmed to be the cause of the intestinal obstruction.

Pre-operatively, the management comprises mainly supportive treatment with IV fluids and decompressive measures in case of obstruction. However, surgical procedure mainly depends on the intraoperative findings. In one of the case reports, the central part of the ileocecal valve was atretic with the valve repair [6]. In our case, we removed the part of the terminal ileum that was dilated and performed an anastomosis between the terminal ileum and the ascending colon with appendectomy. This approach has been adopted by many surgeons in the past and has given satisfactory results. As per the understanding, the part of the colon proximal to the anastomosed bowel should be dissected to prevent the occurrence of ileus postoperatively.

## Conclusions

In conclusion, ileocecal valve atresia is a very rare anomaly with very few cases available in the literature. It commonly presents in neonates with signs of intestinal obstruction. Pre-operatively the diagnosis can be challenging, but imaging studies like plain X-ray abdomen, upper gastrointestinal contrast study, and contrast enema can help with the diagnosis. Intraoperatively, resection of the atretic colon along with ileocolic anastomosis with or without appendectomy has been the accepted management. We, therefore, intend to contribute it to medical literature along with a brief discussion on this rarest topic.
